# Are *Blastocystis hominis* and *Cryptosporidium* spp. playing a positive role in colorectal cancer risk? A systematic review and meta-analysis

**DOI:** 10.1186/s13027-022-00447-x

**Published:** 2022-06-17

**Authors:** Ali Taghipour, Esmail Rayatdoost, Amir Bairami, Saeed Bahadory, Amir Abdoli

**Affiliations:** 1 Zoonoses Research Center, Jahrom University of Medical Sciences, Jahrom, Iran; 2 Department of Emergency Medicine, Jahrom University of Medical Sciences, Jahrom, Iran; 3 Department of Parasitology and Mycology, School of Medicine, Alborz University of Medical Sciences, Karaj, Iran; 4 Department of Parasitology, Faculty of Medical Sciences, Tarbiat Modares University, Tehran, Iran; 5 Department of Medical Parasitology and Mycology, Jahrom University of Medical Sciences, Jahrom, Iran

**Keywords:** *Blastocystis hominis*, *Cryptosporidium* spp., Colorectal cancer, Meta-analysis

## Abstract

**Objective:**

Intestinal protozoa *Blastocystis hominis* and *Cryptosporidium* spp. are two influential factors in intestinal complications and malignancies. In present study, we estimated the pooled prevalence and odds ratio (OR) of the two parasites in colorectal cancer (CRC) patients and their possible association with the deadly disease.

**Method:**

Our systematic search was conducted for published researches between January 1, 2000 and April 30, 2022 by using four international databases include Scopus, PubMed, and Web of Science as well as Google scholar search engine. The random- and fixed-effects models were used to estimate the pooled prevalence, OR, and 95% confidence interval (CI) by comprehensive meta-analysis (V2.2, Bio stat) software. Inclusion and exclusion criteria were applied.

**Results:**

Thirteen papers (seven case–control and six cross-sectional studies) for *B. hominis*/CRC and six papers (two case–control and four cross-sectional studies) for *Cryptosporidium* spp./CRC were eligible to include in data synthesis. Pooled prevalence of *B. hominis* and *Cryptosporidium* spp. in CRC patients was calculated to be 26.8% (95% CI 19.4–35.7%) and 12.7% (95% CI 6.8–22.5%), respectively. Based on case–control studies, significant difference was found between case and controls in both protozoa (*B. hominis* OR 2.10; 95% CI 1.39–3.18% vs. *Cryptosporidium* spp. OR 5.06; 95% CI 1.8–13.6%). Considering the *Blastocystis* subtypes, ST1 (5/6; 83.33% studies) and ST3 (5/6; 83.33% studies) had the highest number of reports in CRC patients. Regarding the *Cryptosporidium* species, only *C. parvum* and *C. hominis* were reported.

**Conclusion:**

Given the significant prevalence of both parasites in CRC patients and their statistically significant association, there is a need to pay more attention to these two intestinal parasites in under treatment patients.

## Introduction

According to the latest figures from the World Health Organization (WHO), 12 million cancerous case and 4 million deaths from cancer have been reported worldwide, of these, colorectal cancer (CRC) rank third in terms of morbidity (~ 2 million) and second in mortality rate (~ 1 million deaths) [1]. Chronic infections and inflammation along with unhealthy diet, stressful lifestyle, cell damages, constant exposure to radiation and harmful chemicals are risk factors for development of cancers [2, 3]. Infectious agents including parasites account for approximately 16% of cancers [4]. As infectious factors, the *Blastocystis hominis* and *Cryptosporidium* spp. are ubiquitous opportunistic protozoa isolated from the human host gastrointestinal tract [5, 6]. These prevalent enteric parasites may cause serious challenges in people undergoing colorectal cancer (CRC) chemotherapy (immunocompromised) due to their location in gastrointestinal tract [4, 7]. Among the *Cryptosporidium* species, *Cryptosporidium parvum* (*C. parvum*) and *Cryptosporidium hominis* (*C. hominis*) are responsible for over 90% of all human cases [8]. So far, out of 22 identified *B. hominis* subtypes (ST1-ST22), ten subtypes have been isolated from humans (ST1-9 and ST12), which ST3 is more prevalent [9]. Both parasites have zoonotic potential and transmission routes are oral-fecal alongside the contaminated water and food sources as well as close animal contact [10, 11]. Although the pathogenesis of *B. hominis* and *Cryptosporidium* spp. have not been clearly established, they have been frequently reported in individuals with gastrointestinal complications including diarrhea, abdominal cramps, etc. [12, 13]. *B. hominis* and *Cryptosporidium* spp. has occasionally been controversially found in healthy people as well as people with gastrointestinal symptoms, the risk of being opportunistic in people undergoing chemotherapy cannot be ignored [14, 15]. Up to now, numerous studies have been conducted on the pathogenic power and possible association of both protozoa with non-communicable diseases such as irritable bowel syndrome (IBS), Crohn's disease, and gastrointestinal cancers [16–19]. In the latter, scattered studies have shed new light on the potential virulence role and prevalence of parasites in CRC. The present systematic review and meta-analysis was designed and performed with the aim of aggregating the available data and providing a comprehensive and statistically documented picture of the pooled prevalence and odds ratio (OR) of *B. hominis* and *Cryptosporidium* spp. in CRC patients and their possible association with the deadly disease.

## Materials and methods

### Search strategy

We followed the preferred reporting items for systematic reviews and meta-analyses (PRISMA) guidelines for the design, analysis and interpretation of the present study [20]. To evaluate the prevalence and OR of *B. hominis* and *Cryptosporidium* spp. in the CRC patients, a search was performed on the related literatures in four international databases, including Scopus, PubMed, and Web of Science as well as Google Scholar scientific search engine between January 1, 2000 and April 30, 2022. The searching process was accomplished using combinations of the following search keywords, including: “*Blastocystis*”, “*Cryptosporidium*” AND “Colorectal cancer”, “Gastrointestibal cancer” in English language.

### Inclusion criteria

The following inclusion criteria were applied in the current review: (1) the peer-reviewed original research papers and short reports; (2) case–control and cross-sectional studies that estimated the prevalence of *B. hominis* and *Cryptosporidium* spp. in CRC patients; and (3) studies published with full text or abstracts in English which published online up to April 30, 2022.

### Exclusion criteria

The exclusion criteria were included: (1) All types of review studies, editorials, letters and case reports; (2) those articles that were not available in English language; and (3) researches that report the prevalence of both parasites in cancers other than CRC, as well as studies with confusing and/or unclear data.

### Study selection and data extraction

The primary screening of eligible studies based on inclusion criteria in mentioned databases was the responsibility of two expert researchers (AT and SB). Additionally, the references of the eligible papers were carefully hand-checked to find relevant articles that were not retrieved in the database searching. After removing duplicate and irrelevant records, and ensuring the existence of extractable data, studies information was extract by the ER and AB. Extracted items included first author name, year of publication, study design, geographical location of study, total CRC patients sample size and the number of isolated *B. homins* and *Cryptosporidium* spp. Finally, the extracted data was double checked by AA and the controversial issues were resolved by AT.

### Quality assessment

To assess the quality of included case–control studies, we used the Newcastle–Ottawa Scale (NOS), as suggested by the Cochrane collaboration [21]. In this 9-star scale, a study could be awarded a maximum of one star for each numbered item within the selection and exposure categories. Also, a maximum of two stars could be given for comparability. Papers with a total score of 0–3, 4–6 or 7–9 points were categorized as poor, moderate or of high quality, respectively. The Joanna Briggs Institute (JBI) checklist also was used for quality assessment of the included cross-sectional records which have contains ten questions with four options including, yes, no, unclear, and not applicable [22]. The papers with a total score of 4–6 and 7–10 points were classified as the moderate and high quality, respectively. We have decided to include (4–10 points) and exclude (≤ 3 points) the researches.

### Data synthesis and statistical analysis

Data was analyzed using comprehensive meta-analysis software version 2. To assessment the association between *B. homins* and *Cryptosporidium* spp. with CRC, an OR and pooled prevalence using random- and fixed-effects models and corresponding 95% confidence intervals (CI) were calculated for each study. In order to assess heterogeneity of studies, *I*^2^ value was considered that the value > 50% indicates a statistical significant heterogeneity. Eggers regression (Qualitative method) was applied to assess the possibility of publication bias during the analysis. *P*-value < 0.05 considered statistically significant.

## Results

### Study characteristics

**Fig. 1:**
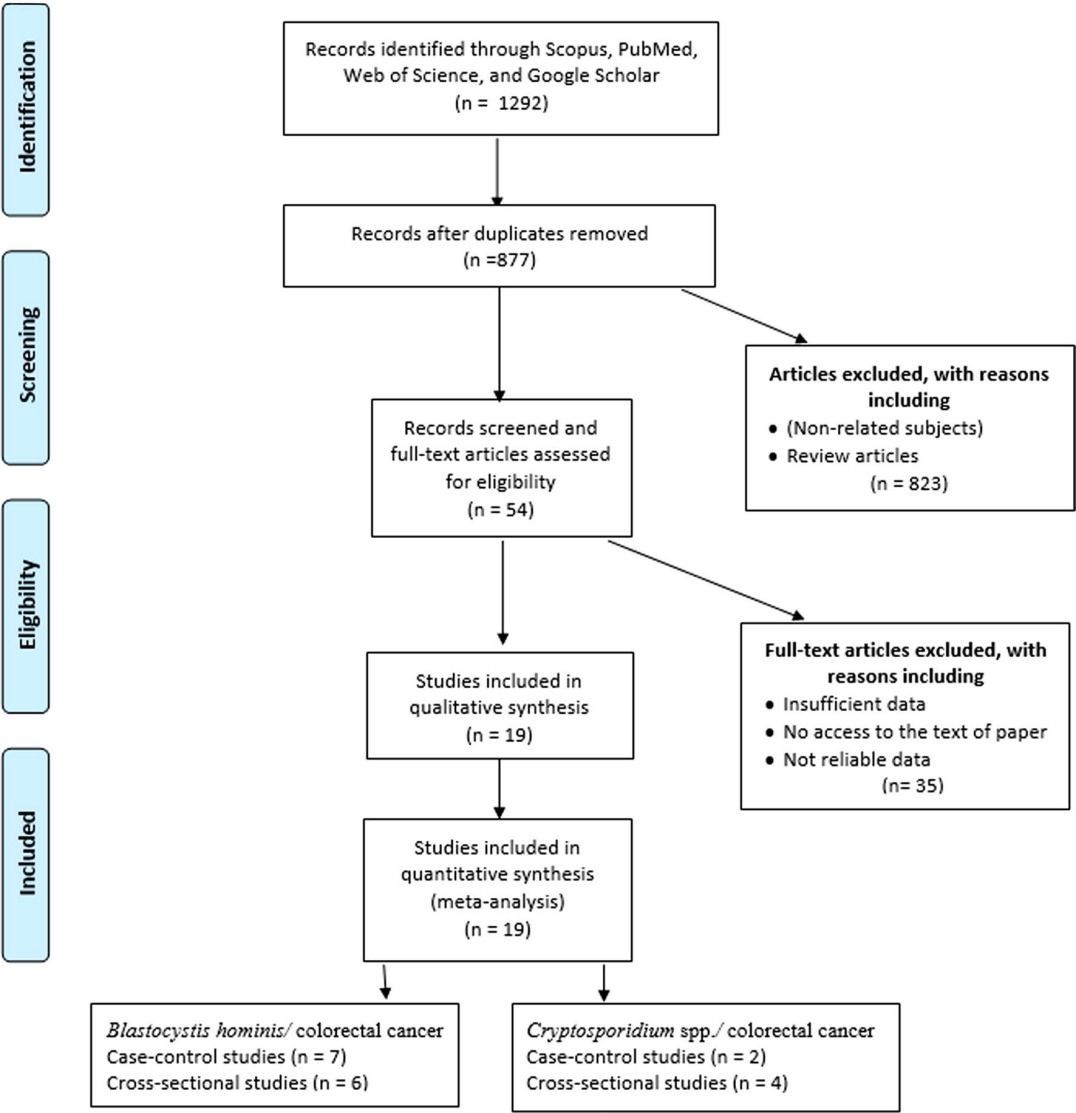
PRISMA flow diagram of the search strategy and study selection process

As shown in Fig. 1, a total of 1292 studies were identified by the initial search in the major databases. Finally, after removing duplicate and papers with non-related subjects, thirteen papers (seven case–control [4, 23–28] and six cross-sectional studies [29–34]) for *B. hominis*/CRC and six papers (two case–control [6, 8] and four cross-sectional studies [35–38]) for *Cryptosporidium* spp./CRC were eligible to include in data synthesis. These studies were conducted in ten different countries from four in Poland (one study for *B. hominis* and three studies for *Cryptosporidium* spp.), three in Iran (only *B. hominis*), two in China (one study for *B. hominis* and one study for *Cryptosporidium* spp.), two in Iraq (only *B. hominis*), two in Malaysia (only *B. hominis*), two in Saudi Arabia (only *B. hominis*), one in Turkey (only *B. hominis*), one in Egypt (only *B. hominis*), one in Tunisia (only *Cryptosporidium* spp.), and one in United States (only *Cryptosporidium* spp.). Further data are shown in Tables 1 and 2. The results of quality assessment according to NOS and JBI for eligible studies are depicted in Tables 1 and 2. The included articles in the present meta-analysis showed an acceptable quality.

**Table 1:** Summary of the included studies reporting prevalence of *Blastocystis hominis* in CRC patients

References	Country	Study design	Diagnostic methods	Statistical significant difference	Total case	Positive case	*Blastocystis* subtypes in case group	Total control	Positive control	*Blastocystis* subtypes in control group	QA
Ali et al. [23]	Egypt	CC*	Microscopic (modified trichrome stain), culture, and PCR	No significant differences (p value = 0.101)	100	52	ST1 (1), ST2 (2), ST3 (4), and ST7 (3)	100	42	ST1 (4), ST2 (2), and ST3 (4)	10
Mahmoudvand et al. [24]	Iran	CC	Microscopic (modified trichrome stain) and PCR	Significant differences (p value < 0.001)	67	16		67	6		9
Hawash et al. [25]	Saudi Arabia	CC	Microscopic	Significant differences (p value < 0.05)	75	20		25	2		9
Sulzyc Bielicka et al. [4]	Poland	CC	Microscopic and PCR	Significant differences (p value = 0.00409)	107	13	ST1 (2), ST2 (1), and ST3 (9)	124	3	ST1 (1) and ST3 (1)	10
Al-Dabbagh and Al-Mukhtar [26]	Iraq	CC	Microscopic (formalin saline) and ELISA (B. hominis antigen)	No significant differences (p value = 0.6956)	40	15		80	33		9
Mohamed et al. [27]	Saudi Arabia	CC	Microscopic, culture, and PCR	Significant differences (p value < 0.05)	74	22	ST1 (12) and ST5 (10)	80	12	ST1 (2), ST2 (7), and ST5 (3)	10
Kumarasamy et al. [28]	Malaysia	CC	Microscopic (formal ether concentration), culture, and PCR	Significant differences (p value < 0.05)	204	43	ST1 (9), ST2 (1), ST3 (26), ST1 + 2 (2), ST2 + 3 (4), and ST5 (1)	221	22	ST1 (6), ST2 (2), ST3 (7), ST2 + 3 (4), and ST5 (3)	10
Asghari et al. [29]	Iran	CS*	Microscopic (modified trichrome stain), culture, and PCR		4	2	ST3 (2)				5
Majeed et al. [30]	Iraq	CS	Microscopic		116	51					7
Esteghamati et al. [31]	Iran	CS	Microscopic and PCR		39	11					6
Zhang et al. [32]	China	CS	Microscopic and PCR		49	4					6
Yersal et al. [33]	Turkey	CS	Microscopic, culture, and PCR		66	5	ST1 (3) and ST3 (2)				7
Chandramathi et al. [34]	Malaysia	CS	Microscopic and culture		15	7					5

*CC: Case–control

*CS: Cross-sectional

QA: Quality assessment

**Table 2:** Summary of the included studies reporting prevalence of *Cryptosporidium* spp. in CRC patients

References	Country	Study design	Diagnostic methods	Statistical significant difference	Total case	Positive case	*Cryptosporidium* species-subtypes in case group	Total control	Positive control	QA
Zhang et al. [6]	China	CC*	PCR	Significant differences (p value < 0.001)	116	20	C. parvum-IIaA15G2R1 (10), C. parvum-IIaA15G2R2 (9), and C. parvum-IIaA13G2R2 (1)	141	0	10
Sulzyc Bielicka et al. [7]	Poland	CC	Immunoenzymatic test	Significant differences (p value = 0.015)	108	14		125	5	10
Essid et al. [35]	Tunisia	CS*	Modified Ziehl Neelsen stain and PCR		15	5	C. hominis-IaA27G1R1 (2), C. parvum-IIaA15G2R1 (1), and C. parvum-IIcA5G3 (2)			7
Shebl et al. [36]	United States	CS	Microscopic		320	7				7
Sulzyc Bielicka et al. [37]	Poland	CS	Immunoenzymatic test		87	11				6
Sulzyc Bielicka et al. [38]	Poland	CS	Enzyme immunoassay		55	10				6

*CC: Case–control

*CS: Cross-sectional

QA: Quality assessment

### B. hominis and CRC

**Fig. 2:**
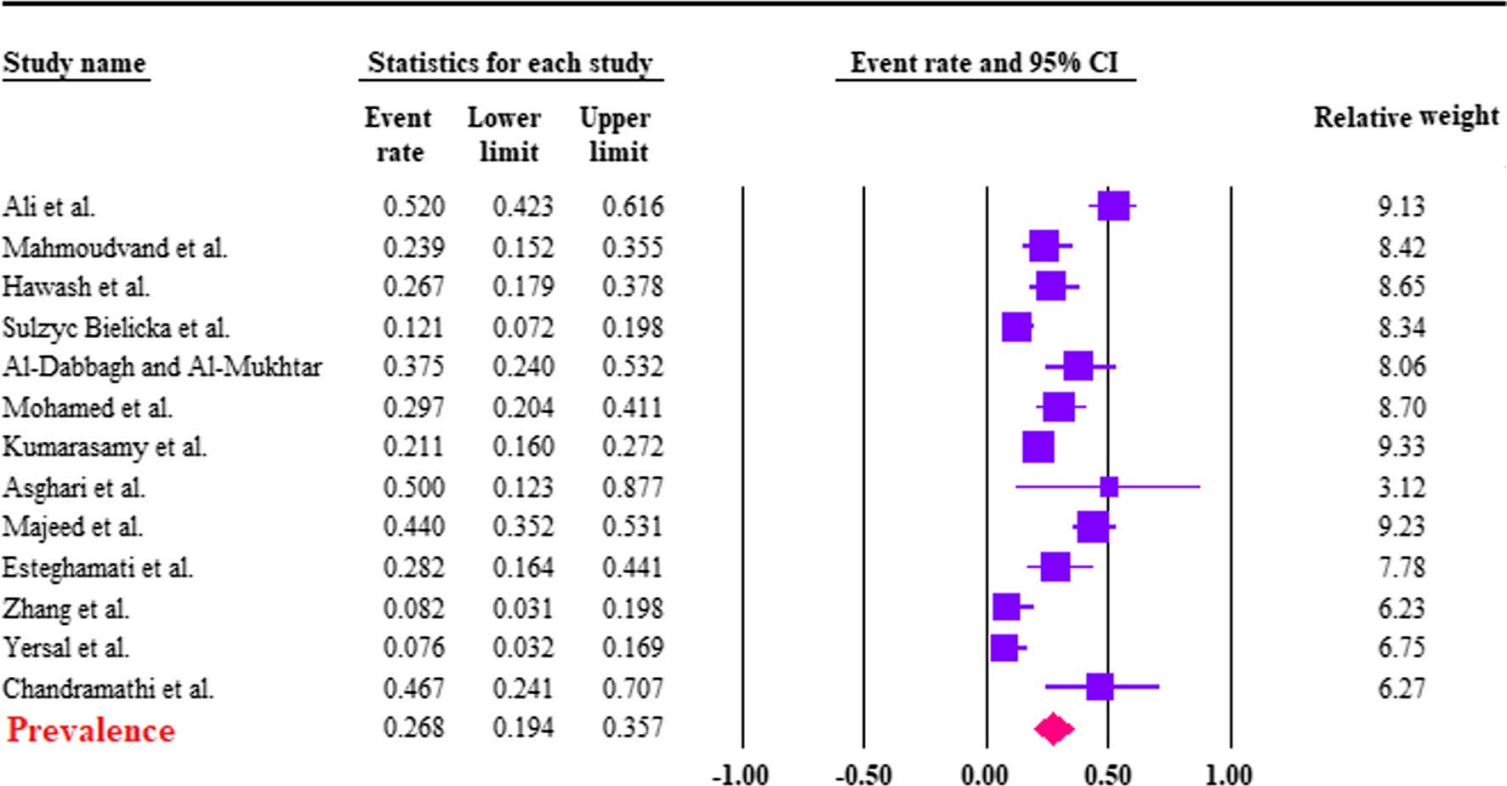
Forest plot of prevalence of *Blastocystis hominis* in CRC patients, estimated with random-effects model

**Fig. 3:**
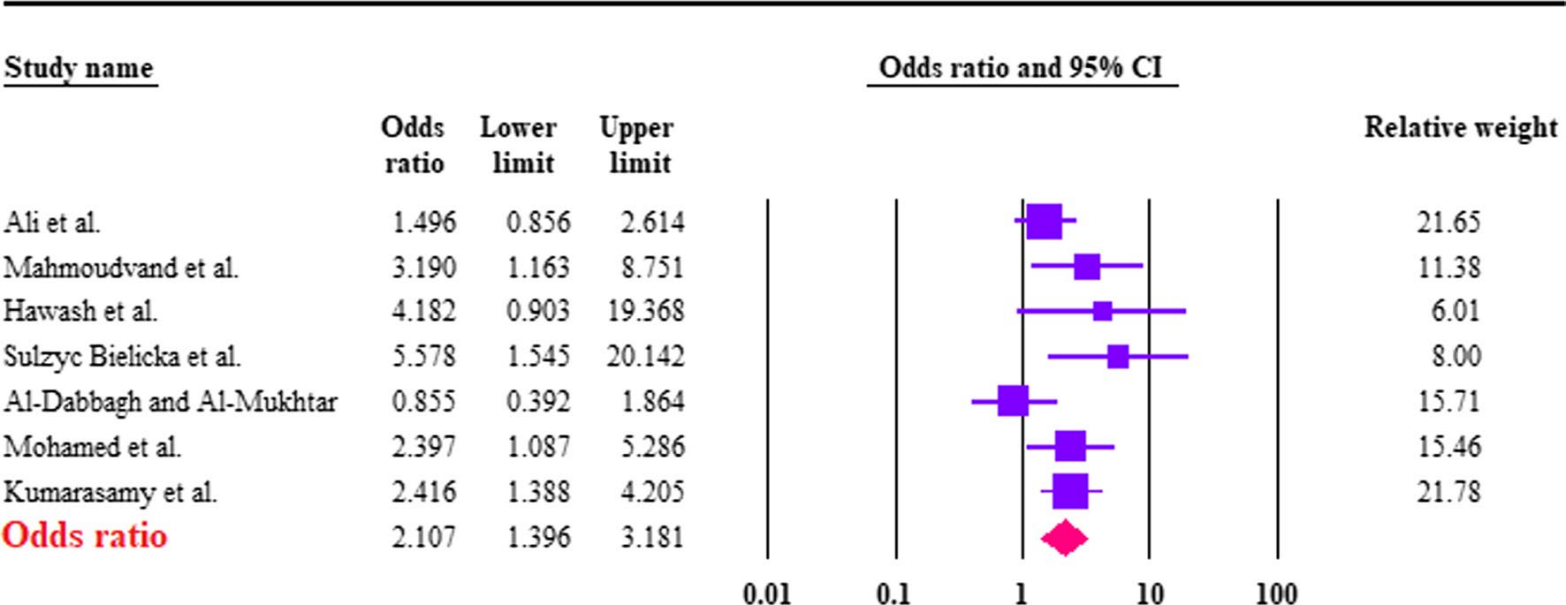
Forest plot of the association between *Blastocystis hominis* and being CRC patients, estimated with random effects model, showing the odds ratio (OR) and 95% confidence interval (CI)

Based on the random-effects model, the pooled prevalence of *B. hominis* in CRC patients was calculated to be 26.8% (95% CI 19.4–35.7%). The heterogeneity was substantial (*I*^2^ = 85.2%; τ^2^ = 0.45; ρ = 0.00). Forest plot diagram is presented in Fig. 2. According to the seven studies with a case–control design, the pooled prevalence of *B. hominis* in case 27.7%; (95% CI 18.8–38.8%; *I*^2^ = 86.8%) was higher than controls 14.4% (95% CI 6.7–28.5%; *I*^2^ = 92.7%), a significant difference was found between case and controls (OR 2.10; 95% CI 1.39–3.18; *I*^2^: 42.4%) (Fig. 3). Six studies had extractable data regarding the *Blastocystis* subtypes. In this regard, ST1 (5/6; 83.33% studies) and ST3 (5/6; 83.33% studies) had the highest number of reports in CRC patients (Table 1).

### Cryptosporidium spp. and CRC

**Fig. 4:**
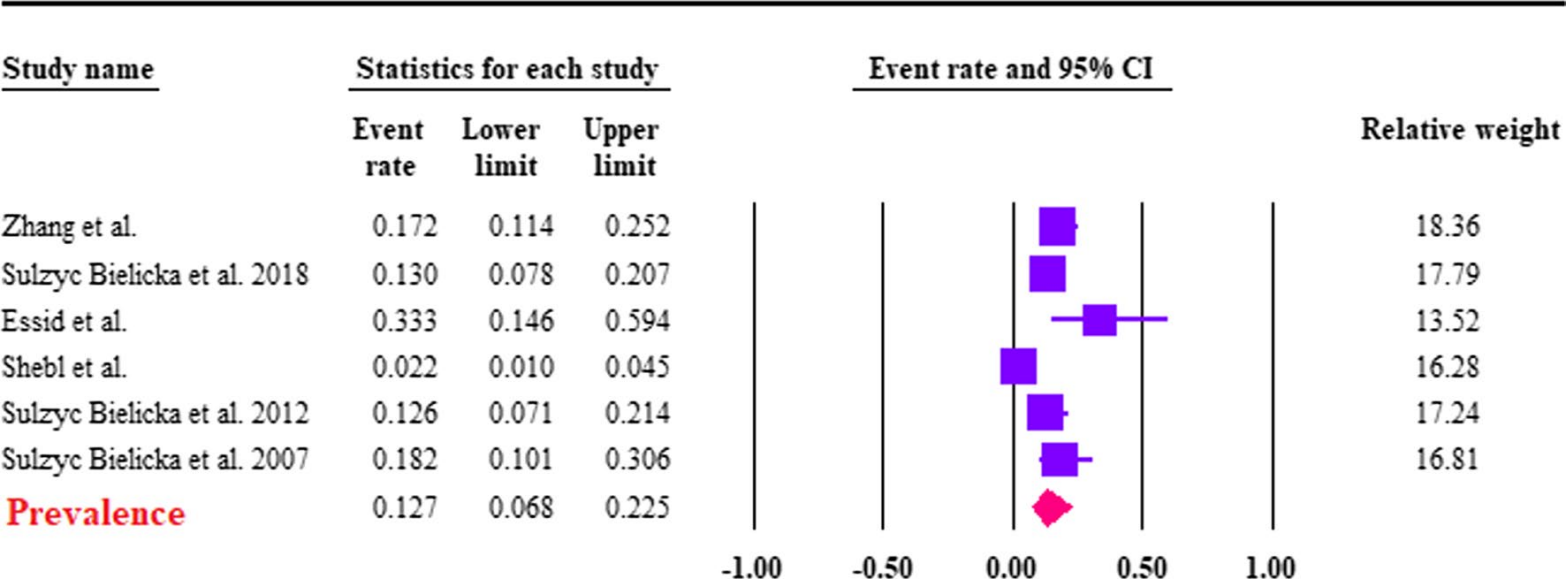
Forest plot of prevalence of *Cryptosporidium* spp. in CRC patients, estimated with random-effects model

**Fig. 5:**
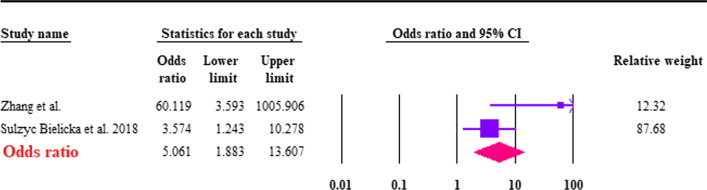
Forest plot of the association between *Cryptosporidium* spp. and being CRC patients, estimated with fixed effects model, showing the odds ratio (OR) and 95% confidence interval (CI)

The estimation of the pooled prevalence of *Cryptosporidium* spp. was 12.7% (95% CI 6.8–22.5%; *I*^2^ = 84.7%) among CRC patients (Fig. 4). Based on the two case–control studies, we found that the pooled prevalence of *Cryptosporidium* spp. was significantly higher in CRC patients 15.3% (95% CI 11.1–20.7%; *I*^2^ = 80.2%) compared to controls 1.7% (95% CI 0.2–15%; *I*^2^ = 88.1%), the difference between the case and control groups was significant (OR 5.06; 95% CI 1.8–13.6; *I*^2^: 70.4%) (Fig. 5). *Cryptosporidium* species were identified in only two studies. Among them, only *C. parvum* and *C. hominis* were reported; the type of their subtype is shown in Table 2.

### Publication bias

Considering the *B. hominis* and CRC studies, detecting publication bias using the Eggers regression revealed that publication bias in case–control studies was not statistically significant (*p* value < 0.05). Due to the fact that only two case–control studies were performed on *Cryptosporidium* spp. and CRC, publication bias is not applicable.

## Discussion

Over the past decade, there has been considerable evidence of parasitic infections, such as *Cryptosporidium* spp. and *B. hominis*, with various types of cancer [39, 40]. *Cryptosporidium* spp. and *B. hominis* have been suggested as important intestinal parasites in CRC patients and the severe form of these diseases occurs most frequently in such patients [8, 41]. In this regard, the current study is a systematic review and meta-analysis to address the pooled prevalence and ORs of *Cryptosporidium* spp. and *B. hominis* infections in CRC patients compared with non-cancer individuals. This meta-analysis revealed a positive association between *Cryptosporidium* spp. and *B. hominis* infections with CRC. Among the seven case–control studies regarding the *B. hominis*, five reported a significant and two reported a non-significant difference related with *B. hominis* infection in the case group compared to the control group (Table 1). Both case control studies on *Cryptosporidium* spp./CRC showed a significant prevalence of this protozoan in the case group compared to the control group (Table 2). High heterogeneity (*I*^2^) was observed in this meta-analysis. Several sources of heterogeneity have been reported in the literature which include study design, detection method, geographical distribution, sample size, and high or low prevalence in some studies (different weights of each study) [42, 43].

Considering *B. hominis*, some studies conducted on in vitro and in vivo, and epidemiological studies from human populations have revealed an association between *B. hominis* and CRC [27, 41]. In this regard, in vitro studies have shown severe cytopathic and immunological effects by the solubilized antigen of *B. hominis* in human colorectal cancer cell line [41]. The findings of these studies suggest that *B. hominis* infection may increase the proliferative, invasive, and metastatic properties of CRC cells [41]. Another in vitro study indicated that the five subtypes of *B. hominis* significantly increased the proliferation of human CRC cell line HCT116, particularly ST3 [44]. The present systematic review has shown that ST1 and ST3 is more common in CRC patients than other subtypes. It is suggested that in the future studies, in order to deeper understanding the mechanism of these subtypes in CRC development, further cellular studies should be performed focusing on these subtypes in CRC patients.

Regarding the *Cryptosporidium* spp., several experimental and epidemiological studies have shown the potential role of cryptosporidiosis and CRC progression [19, 38, 45]. It has been suggested that *C. parvum* is one of the pathogen agents that may trigger intestinal dysplasia [45]. However, the pathophysiological mechanisms of *Cryptosporidium* spp. infection are multifactorial and not completely specified. An experimental study revealed that *C. parvum* is able to modulate host-cell cytoskeleton and intracellular signals, which may explain the transformed phenotype of the infected epithelial cells [46]. Moreover, our findings showed that several *C. parvum* (IIa and IIc) and *C. hominis* (Ia) subtypes were present in CRC patients (Table 2). Therefore, it is suggested to evaluate the progression of CRC in laboratory and human studies by considering these species/subtypes.

Some limitations of this systematic review and meta-analysis, which may affect the results, are listed as follows: (1) lack of access to the full text of some articles, (2) low sample size of some studies, (3) geographical dispersion of studies, (4) different diagnostic methods and (5) lack of some variables such as age and gender. Also, the state of immunosuppression of the patients and the time of the evolution of the CRC may be another source of heterogeneity which was not considered in the present study.

In conclusion, the present meta-analysis demonstrates that CRC may be related with elevated risks of *Cryptosporidium* spp. and *B. hominis* infections. However, further studies should be performed to investigate the impact of *Cryptosporidium* spp. and *B. hominis* infections in the onset or development of CRC in the future.

## Data Availability

The data used to support the findings of this study are included within the article.
